# Associations Between Coping Strategies and Gambling Disorders in University Students: An Exploratory Neural Network Study

**DOI:** 10.3390/bs16040564

**Published:** 2026-04-09

**Authors:** José Miguel Giménez-Lozano, Francisco Manuel Morales-Rodríguez, Juan Pedro Martínez-Ramón

**Affiliations:** 1 Department of Developmental and Educational Psychology, Centro Enseñanza Superior ESCUNI, 28047 Madrid, Spain; 2 Department of Developmental and Educational Psychology, University Campus of Cartuja, University of Granada, 18071 Granada, Spain; fmmorales@ugr.es; 3 Department of Developmental and Educational Psychology, Campus of Excellence Mare Nostrum, University of Murcia, Espinardo, 30100 Murcia, Spain; juanpedromartinezramon@um.es

**Keywords:** artificial neural networks, coping strategies, gambling disorders, university students

## Abstract

Background: Gambling disorders are an escalating public health issue, with notable increases across age groups, particularly among adolescents and young adults. This study examines the role of coping strategies in gambling behaviors among university students aged 17–48 years and explores the prediction potential of artificial neural networks. Methods: The sample included 218 participants (*M* = 21.89, *SD* = 5.57). Results: A multilayer perceptron neural network was implemented to classify gambling risk based on coping strategies. Significant correlations between specific coping strategies and higher levels of gambling disorders were revealed. The neural network model demonstrated an 85% accuracy in predicting gambling risk, with the most influential factors identified as autonomy, negative urgency, gender, denial, and lack of perseverance. Conclusions: These findings highlight the effectiveness of neural networks in identifying individuals most at risk for GDs.

## 1. Introduction

### 1.1. Current Situation of Gambling Disorders

Gambling disorders (GDs) are currently classified as a behavioral addiction—that is, an addiction not involving substances—in the main diagnostic systems, including the DSM-5-TR (APA, 2022). In this regard, GDs involve loss of control, the progressive prioritization of gambling over other activities, and persistence despite adverse consequences for the individual’s mental and physical health, increasing financial debt and representing a rise in risk factors associated with aggressive behavior (García-Maza et al., 2025). Indebtedness, one of the most serious harms associated with GDs, has been linked to gambling-related suicide (Andreeva et al., 2022). For example, a retrospective cohort study conducted by Karlsson and Håkansson (2018) involving 2000 individuals with GDs in Switzerland found a mortality ratio 1.8 times higher than that observed among those who had not been diagnosed with this disorder. This ratio increased to 6.2 among young adults aged 20 to 49 years.

Among adults in the general population, the estimated global prevalence of gambling in the previous 12 months is 46.2%, with higher participation rates among men (49.1%) than among women (37.4%). One investigation estimates that the current global gambling environment may adversely affect approximately 448.7 million adults, including around 80 million adults experiencing gambling disorder or problematic gambling. It also warns that the commercial gambling market is expanding rapidly, with consumer net losses projected to approach 700 billion dollars by 2028. This expansion is closely tied to digitalization, financial technology, algorithmic targeting, and the convergence between gambling, sports, and online media ecosystems (Wardle et al., 2024).

A meta-analysis by Gabellini et al. (2023) of recent adult prevalence studies published between 2016 and 2022 found pooled estimates of 2.43% for moderate risk or at-risk gambling and 1.29% for problem pathological gambling. However, these rates differed substantially depending on the screening instrument used and the mode of data collection. Studies using the PGSI tended to yield lower estimates than studies using other tools and face-to-face interviews generally produced lower prevalence rates than telephone or online surveys. This methodological variability is crucial when comparing countries, age groups, or temporal trends, and it should be explicitly acknowledged in scientific writing to avoid overinterpreting raw prevalence contrasts.

According to the report based on ESTUDES (2023), 21.5% of students aged 14–18 years had engaged in gambling (online and/or land-based) in the previous 12 months. Participation was substantially higher among boys (29.4%) than girls (13.3%). Land-based gambling remained more prevalent than online gambling (17.7% vs. 10.7%), although digital gambling remains the main source of concern because of its addictive potential and its convergence with broader digital lifestyles.

### 1.2. Gambling Disorders and University Students

University students therefore constitute one of the main at-risk groups for the development of GDs. During the university stage, students face a period of life changes accompanied by new and stressful experiences inherent to higher academic demands, which may increase their susceptibility to engaging prematurely in gambling behaviors and, subsequently, to developing problems that may escalate into GDs (Scandroglio et al., 2023).

Evidence from a cross-sectional study with university students in Turkey showed that gambling was a common behavior in this population: 41.4% reported lifetime gambling, 21.5% reported gambling in the last month, and 15.3% reported gambling online. While pathological gambling was identified in only 1.2% of the sample, the high proportion of students with gambling experience highlights the relevance of this behavior in university settings (Vayısoğlu et al., 2019). Another study (Esparza-Reig et al., 2023) conducted with 258 university students found that gambling addiction was associated with a range of clinical, social, and cognitive variables. Cognitive distortions emerged as a central explanatory factor, and the proposed models accounted for between 16.8% and 34.5% of the variance in gambling addiction. These findings suggest that gambling-related problems in university students are not only linked to emotional and behavioral factors, but also to maladaptive beliefs and cognitive processes, which are especially relevant for prevention and intervention efforts.

### 1.3. Coping Strategies

Lazarus and Folkman (1986), leading researchers on coping strategies, defined coping as cognitive efforts impacting behavior aimed at managing both external demands (e.g., environmental stressors) and internal demands (particularly emotional states). They explained that stressful situations prompt three key processes associated with emotional processing: (1) double assessment, where an individual assesses risk, consequences, and personal coping ability; (2) associated emotions, involving a self-evaluation of emotions and possible outcomes; and (3) behavioral efforts, estimating the necessary effort and success probability in coping with the stressor. They distinguished between two main types of coping strategies: problem-focused coping, aimed at modifying the stressor itself, and emotion-focused coping, aimed at regulating emotional responses.

Coping strategies encompass the cognitive and behavioral efforts individuals deploy to manage internal and external demands that are perceived as taxing or exceeding their resources (Hatunoglu, 2020). University students frequently encounter elevated stress levels, which can influence their coping responses. When healthy coping mechanisms are limited, students may resort to maladaptive strategies, including substance use, to alleviate stress (Böke et al., 2019).

The relationship between coping styles and gambling behaviors is well-documented. Adolescents who engage in excessive gambling often exhibit coping styles characterized by emotion-based, avoidant, and distraction-oriented approaches (Gupta et al., 2004). These coping motivations for gambling also predict future problem gambling behaviors over time, even when controlling for baseline gambling engagement and personality traits like neuroticism (Komoto, 2025). For example, a longitudinal study identified robust associations between coping motivations, positive gambling expectations, and problem gambling (Grubbs & Rosansky, 2020).

Furthermore, family dynamics can indirectly influence youth gambling through coping mechanisms. Attachment to parental figures, particularly dimensions such as angry distress, can exert an indirect effect on problematic gambling via emotion-focused coping styles. This suggests that youth gambling is a multifaceted phenomenon, where family variables interact with individual coping strategies to predict behavioral outcomes (Calado et al., 2017). The findings underscore the utility of teaching high-risk youth more effective, active coping strategies as a foundational component of school-based prevention initiatives aimed at minimizing engagement in addictive behaviors (Gupta et al., 2004).

### 1.4. GD Prediction Using Artificial Neural Networks

Artificial neural networks (ANNs), a subset of machine learning (ML), are powerful tools for addressing various challenges in addiction research, including identifying individuals with substance use disorders, discerning patterns in neuroimages, and understanding prognostic factors (Cresta Morgado et al., 2022). ANNs function as a supervised learning method, capable of identifying complex, non-linear relationships within data. The application of ML and AI in addiction medicine holds considerable promise for achieving early and precise identification of addiction-related issues, potentially revolutionizing therapeutic approaches (Poudel et al., 2024).

Regarding GDs, ANNs and other ML algorithms can effectively detect at-risk users, particularly in the context of online gambling, where activities are increasingly migrating. By leveraging demographic and behavioral data from online gamblers, ML models can classify individuals at moderate-to-high or high risk for gambling-related harms with notable accuracy. For instance, models achieved Area Under the Receiver Operating Characteristic Curve (AUC) scores of 83.20% for moderate-to-high risk (PGSI 5+) and 87.70% for high risk (PGSI 8+) in a study involving French online gamblers. These predictive models also uncovered novel behavioral markers associated with harmful online gambling (Kairouz et al., 2023).

The utility of such algorithms extends to developing targeted harm prevention strategies and facilitating referrals for treatment for at-risk individuals by gambling operators and regulators. However, the successful deployment of AI in addiction medicine, including for gambling disorders, is not without challenges. These include the requirement for extensive datasets for training robust models and the susceptibility of models to overfitting. Innovative strategies, such as integrated transfer learning and multitask learning within Graph Neural Networks (GNNs), have shown promise in enhancing predictive accuracy and robustness even with smaller datasets, thus addressing some data limitations (Xiao et al., 2024). The continued refinement of these computational approaches holds promise for advancing the understanding and intervention of GDs.

### 1.5. Aim and Hypotheses

The aim of this study was to examine the relationship between coping strategies and gambling disorders in university students, and to evaluate the predictive capacity of an artificial neural network. We hypothesized that maladaptive coping strategies would be positively associated with gambling severity and that the model would achieve moderate predictive accuracy.

## 2. Methodology

### 2.1. Procedure

In terms of design, this study employed a quantitative, cross-sectional, ex post facto design approach. A non-probability snowball sampling method was conducted to contact potential participants by sending a description of the study objective and a link to an online survey in periodic waves, as described by Morales-Rodríguez (2021). This study was conducted between September 2021 and April 2022. Regarding the ethical aspects of the research, the guidelines of the Declaration of Helsinki were considered throughout the study, and it was approved by an Ethics Committee of the University of Granada. Informed consent was required.

Participation in the study was voluntary, anonymous and confidential.

With respect to the procedure, participant recruitment was conducted in two phases. In the first phase, we contacted university instructors—either by e-mail or in person, depending on the university and faculty—to request access to their students. In this initial approach, instructors were informed about the study objectives and the research procedure, which consisted of distributing a link to an online survey containing the full set of questionnaires.

Before accessing the questionnaires, students who agreed to participate were presented with a privacy statement describing their data protection rights and were required to indicate their acceptance to proceed. They were also provided with an informed consent form, which likewise required affirmative agreement before continuing with the battery of measures.

### 2.2. Participants

The study had *n* = 218 participants; 70.64% were female (*n* = 154) and 29.36% (*n* = 64) were students from the Spanish universities of the cities of Granada (58%, *n* = 126) and Valencia (33.67%, *n* = 82), and the University of Murcia (7.56%, *n* = 10). Inclusion criteria included being enrolled in a university degree program and being over 18 years old; incomplete responses were excluded. The total sample consisted of subjects with a chronological age ranging from 17 to 48 years. The mean age of the participants was 21.89 (SD = 5.57). The largest age group was 18 years (28%, *n* = 61), followed by 19 (14.7%, *n* = 32), which was very closely followed by 20 (10.6%, *n* = 23) and 22 (10.1%, *n* = 22). As can be seen, the age group younger than 30 years was the majority, practically occupying 90% of the sample. At later ages, we observed residual percentages. Most of the participants came from the Faculty of Educational Sciences (68%, *n* = 148), of which, 54.6% (*n* = 80) were in the Speech Therapy program, 34.47% (*n* = 50) were in the Primary Education program, and to a lesser extent, there were students from the Early Childhood Education program (*n* = 8), the Pedagogy program (*n* = 4) and the Social Education program (*n* = 4). The remaining participants studied at the Faculty of Psychology (*n* = 70). Regarding the year of the program, the vast majority were in their first year of the degree (83.27%, *n* = 181), followed by second year (16.97%, *n* = 37).

### 2.3. Instruments

Standardized and previously validated questionnaires were administered for this study. They are described in more detail below.

Gambling Symptom Assessment Scale (G-SAS). Gambling symptom severity over the previous week was assessed using the G-SAS, a 12-item self-report measure developed to quantify symptom severity and change over time in individuals with a gambling disorder/GD (primarily adult clinical samples). The G-SAS is not intended as a diagnostic or screening instrument. For this reason, the instrument was used as a continuous measure of severity rather than as a diagnostic criterion. Items are rated on a 0–4 scale and refer to average symptoms during the past 7 days. The content covers gambling urges (items 1–4), gambling-related thoughts (items 5–7), time spent gambling or in gambling-related activities (item 8), anticipatory tension/excitement (item 9), excitement/pleasure when winning (item 10), emotional distress (item 11), and personal trouble/impairment (item 12). Total scores range from 0 to 48, with higher scores indicating greater symptom severity; the suggested severity bands are mild (8–20), moderate (21–30), severe (31–40), and extreme (41–48). In the original validation study, the scale showed good internal consistency (Cronbach’s α ≈ 0.87).

Coping strategies. Coping strategies were assessed using the Modified Coping Strategies Scale (Escala de Estrategias de Coping Modificada, EEC-M), which was developed and psychometrically validated by Londoño et al. (2006) for Spanish-speaking populations. The instrument was derived from the revised Coping Strategies Scale and was designed to evaluate cognitive and behavioral responses to stress. Although the original pool comprised 98 items, the final validated version consists of 69 items rated on a 6-point Likert-type scale ranging from 1 (never) to 6 (always). The questionnaire assesses 12 coping dimensions: problem solving, social support, waiting, religion, emotional avoidance, search for professional support, aggressive reaction, cognitive avoidance, positive reappraisal, expression of coping difficulty, denial, and autonomy. Higher scores indicate greater use of that coping strategy. In the original validation study, the final version showed adequate internal consistency, with a global Cronbach’s alpha of 0.847 (Londoño et al., 2006).

### 2.4. Data Analysis

Regarding data analysis, a descriptive analysis of the main dispersion and central tendency indexes was performed. An inferential analysis was also performed. Within the inferential analysis, three artificial neural networks (ANN1, ANN2 and ANN3) were established based on a backward information propagation model and three layers (input, hidden and output) to form a multilayer perceptron network, which was trained with the objective of correctly classifying cases. The cases were distributed in such a way that the approximate proportion chosen by the network tended to be 70% for the training phase, 15% for the testing phase and the remaining 15% was used to perform a predictive analysis of new cases that were not used for the development of the algorithm. We followed the statistical procedure suggested in the study by Morales-Rodríguez et al. (2023). A multilayer perceptron with one hidden layer was trained using the Adam optimizer. The SPSS statistical package (version 28) was used.

## 3. Results

Table 1 shows the results (%) based on the final score and the corresponding classification of gambling risk, obtained using the G-SAS questionnaire. It should be noted that only 0.1% of the sample showed some level of problem gambling (scores of 8–10). Moderate gamblers were those who obtain a score between 3 and 7, with an average of 0.5% within this range.

To test the association between the different factors that make up the coping strategies and moderate or problematic cases of gambling risk based on the G-SAS criteria, we first performed Spearman correlation between the total scores of each of the factors covered by the EEC-M questionnaire and the total score obtained from the G-SAS for each of the participants.

As can be seen in Table 2, small-to-moderate positive correlations were observed between maladaptive coping strategies and gambling severity. The coping strategies with a positive sign—which indicates that if one variable increases, the other would also increase—were religion (r = 0.284; *p* < 0.00), emotional avoidance (r = 0.309; *p* < 0.00), expression of coping difficulty (r = 0.267; *p* < 0.01), denial (r = 0.258; *p* < 0.00) and autonomy (r = 0.286; *p* < 0.00). The strategy with negative correlations—that is, if one variable decreases, the other variable would increase—was waiting (r = −0.303; *p* < 0.00). Table 2 shows the results of the correlations between the G-SAS criteria and the coping strategies (EEC-M).

In Table 3 (ANN classification), it is possible to analyze the percentage correctness of the ANN in predicting case outcomes. The percentage of correctness went up as the ANN stages progressed such that, from the training phase to the testing phase, there was an increase of approximately 3% and from the testing phase to the last reserve phase, it increased by 7%. At this point, it is necessary to explain the significance of each of these phases. Thus, in the training phase, the network randomly selects the previously established percentage of the participating sample and, by means of a retroactivation algorithm, analyzes the answers given in the DV and which were the answers given in the VI and which are associated with affirmative or negative answers (0 or 1) in the DV. In the testing phase and once the ANN has generated its predictive algorithm, it tests whether this algorithm can predict the cases that are presented in the aforementioned percentage of representation. In this case, the network continues to adjust and learn to refine its algorithm. Finally, in the reserve phase, it selects cases that it has not previously handled and tries to predict their response in the DV based on their scores in the VI using the algorithm perfected in the testing phase, without making any modifications to it. In this way, it is possible to observe how the predictive ability of the ANN changes as it progresses through training and testing and is finally tested in the reserve phase.

**Figure 1:**
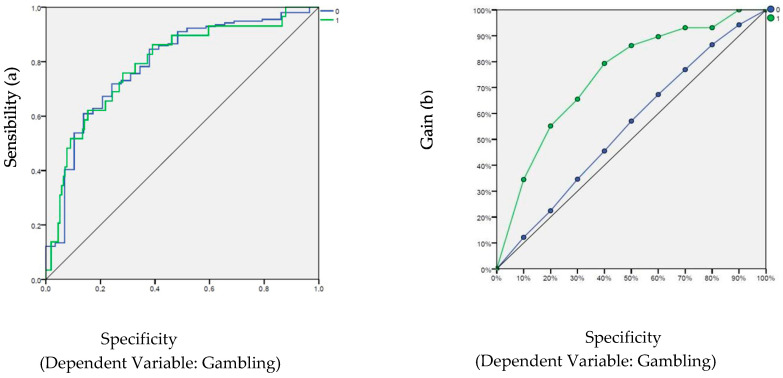
ROC curves for mobile gambling (VD) sensibility (**a**) and gain (**b**).

To evaluate the predictive and discriminative capacity of the model created for this project, we looked at the ROC curves (see Figure 1). ROC plot curves show the relationship between sensitivity and false positives (complementary to specificity) for different decision thresholds. Any curve on the left side of the diagonal has a probability greater than chance. In this case, both the probability of correctly predicting the cases that scored 0 and the probability of correctly predicting the cases that scored 1 on the dependent variable is greater than 0.5. Specifically, the area above the diagonal (or the probability of the curve) was 0.79, which suggests a model with moderate-to-high discriminative capacity, an appropriate level for behavioral variables such as the dependent variable gambling. The ROC curves consistently lie well above the non-discrimination line, which is represented by a 45-degree diagonal, thus exceeding the threshold of chance. In gambling research, class imbalance may occur, and ROC curves help reveal the sensitivity. A high sensitivity is necessary for the model to detect individuals at high risk. In Figure 1, the people who scored 1 (did gamble with a cell phone) had different behavior than those with a value of 0 (did not gamble with a cell phone).

The SPSS program made it possible for us to evaluate the importance of each variable in the model. Table 4 shows the values obtained from the analysis of the weights of each network connection presented above. Table 4 below shows, on a scale from 0 to 1 (importance) or on a scale from 0 to 100 (normalized importance), the contribution of each independent variable to the neural model.

It can be observed that autonomy was one of the most important variables in predicting gambling behavior in 100% of the cases; the second best predictor was negative urgency (91%) and the third was sex (86.4%). We found that denial predicted gambling behavior in 80.80% of cases, and very close behind was positive re-evaluation (73.90%). Between the 50–60 percentile were the coping strategies of seeking professional support (57%) and cognitive avoidance (56%). The rest of the variables were below the 50% percentile and their predictive power could be questioned.

## 4. Discussion

This study sought to explore the association between coping strategies and gambling disorder among university students, as well as to assess the predictive performance of an artificial neural network. In line with our expectations, maladaptive coping strategies were anticipated to show a positive relationship with gambling severity, and the model was expected to demonstrate a moderate level of predictive accuracy.

As already indicated in Section 3, it is observed through the G-SAS questionnaire that only 0.1% of the sample showed some level of problem gambling and 0.5% showed moderate gambling. The model exhibits high specificity and overall accuracy in identifying non-risk individuals but low sensitivity for the positive class. This indicates that the model may have value as a conservative screening tool or initial filter, particularly when minimizing false positives is a priority. However, its limited sensitivity should be taken into account when considering practical applications. Anyway, if we consider that the studies conducted to date, in Spain, problem gamblers only comprise 0.3% of the population (Gómez-Yáñez, 2017).

Another study estimated that 1.8% of people can be categorized as problem gamblers (ADSIS Foundation, 2022). They pointed out that 12% of the people interviewed presented indicators of addiction to sports betting, such as feeling guilty when placing a bet and/or because of the consequences or having tried to quit without being able to do so. Another 10% admitted to having problems with gambling. But this does not mean that they had a serious problem that could be diagnosed as problem gambling. There are other data or factors (related to gambling), but they must be observed in isolation and must be studied in conjunction with other indicators to be considered as such. This same report estimates, through a systematic review of several studies, that Spain is one of the countries with the lowest rate of problem gambling among its population. For example, one of the most important regions of China, Macao, has a problem gambling prevalence of 4.3%, while in South Africa, it is 3.0%, and within Europe, Iceland is one of the countries with the highest rate at 1.6%.

Avoidant and escape-oriented coping strategies are consistently associated with addictive behaviors. Recent evidence indicates that attempts to manage stress or negative affect through avoidance may increase vulnerability to both substance-related and behavioral addictions. In the gambling field, the current evidence shows that gambling motivated by escape from distress is a significant correlate of problem gambling and may contribute to its onset and maintenance. This supports the view that addictive behaviors can function as maladaptive attempts to regulate aversive internal states rather than solely as responses to the rewarding properties of substances or gambling itself (Alaba-Ekpo et al., 2025).

A similar pattern has been documented in the alcohol research. Recent findings show that avoidant coping is positively associated with problematic alcohol use, particularly among trauma-exposed individuals and those with post-traumatic stress symptoms. Avoidant coping is therefore better understood not merely as a correlate, but as a clinically relevant risk mechanism linking depressive symptoms, trauma-related distress, and alcohol misuse (Danielson et al., 2024). More broadly, the literature continues to show that maladaptive coping in stressful contexts is associated with poorer mental health outcomes, including depression and post-traumatic stress symptoms (Danielson et al., 2024).

In the present study, higher gambling severity was associated with greater use of avoidant coping, as well as with difficulties in coping expression and emotion-related processing. The current evidence on addictive disorders is increasingly emphasizing that deficits in emotion regulation—especially limited emotional awareness, poor identification of emotions, and difficulty expressing distress—are clinically relevant. These processes may interfere with help-seeking, reinforce denial, and maintain gambling behavior as a short-term strategy for affect regulation. For this reason, contemporary interventions are increasingly targeting emotion regulation directly, including focusing on emotional awareness, identification, and expression, alongside more traditional cognitive-behavioral components (Chrétien et al., 2025).

The association between gambling and religion or religiosity should be interpreted with caution. Recent evidence suggests that religiosity is not a straightforward risk factor; rather, its role appears to be context-dependent. In some cultural settings, religiosity may operate as a protective factor against problematic gambling, whereas in others, it may indirectly relate to gambling through cognitive distortions such as illusion of control or interpretative bias. Consequently, a more nuanced interpretation is warranted: rather than equating gambling with religion, it is more accurate to examine how belief systems, perceived control, and meaning-making processes shape gambling-related cognitions and behaviors (Calado et al., 2024).

Finally, recovery from addictive behaviors typically requires more than merely acknowledging the existence of a problem; it also involves developing more adaptive coping responses and improving the capacity to communicate distress effectively. In this sense, difficulties in expressing negative emotions or asking for support may perpetuate both substance use and gambling-related problems. Contemporary treatment models are therefore placing increased emphasis on strengthening emotional insight, interpersonal communication, and adaptive coping skills as key elements of prevention and recovery (Chrétien et al., 2025; Danielson et al., 2024).

Binde (2007) posited that religiosity might increase gambling participation, as religious people often rely on a favorable outcome through a higher entity. Gamblers with religious beliefs might perceive that a divine force will assist them in maximizing their gambling achievements, even when the objective odds of success are unfavorable. In short, religiosity could make people more prone to falling for gambling-related fallacies. Therefore, the individuals who have these types of thoughts (e.g., the belief that a lucky person can positively influence the odds of success) would have an easier time suffering from a problem with gambling, which is considered a risk factor (Wohl & Enzle, 2002). Kim et al. (2006), in the study they conducted, found that coping strategies such as religiosity were associated with the so-called gambler’s fallacies and problem gambling, and these beliefs were a very important mediator between both variables. In summary, and according to the current research, the association between religiosity and gambling may reflect cognitive distortions or beliefs in external control, although further empirical research is needed.

With regard to the neural network developed in this study and its potential to predict gambling disorders (GDs), sex was found to be a relevant variable, but this should not be treated as sufficient evidence on its own. Contemporary evidence continues to show that men often present higher-risk gambling profiles in many samples; however, predictive studies also indicate that demographic variables do not show uniform effects across datasets. For instance, in recent machine-learning research based on account-level gambling data, age showed a clearer association with self-reported problem gambling than gender, and gender did not consistently differentiate problem gamblers from non-problem gamblers. Therefore, the higher gambling scores observed among men in the present sample are better interpreted as part of a broader multivariate risk pattern rather than as evidence of a simple causal sex effect (Hopfgartner et al., 2024; Krupka et al., 2025).

In the present study, the comparison of means between men and women across the dimensions of coping revealed significant differences in search for social support, waiting, emotional avoidance, positive reappraisal, expression of coping difficulty, and autonomy. Women scored higher in search for social support and autonomy, whereas men scored higher in waiting, emotional avoidance, positive reappraisal, and expression of coping difficulty. These findings should nevertheless be interpreted cautiously because statistical significance does not necessarily imply a large practical difference, especially when the absolute distance between group means was small (Rizzo et al., 2023). More importantly, coping strategies are not inherently adaptive or maladaptive in all circumstances; rather, their function depends on the context and the rigidity with which they are used. Even so, recent evidence in adolescent and young-adult gambling suggests that greater gambling severity is more consistently associated with emotion-oriented and avoidance-oriented coping than with task-oriented or problem-focused coping, which supports interpreting sex differences through the lens of stress regulation and emotional management rather than sex alone (Alaba-Ekpo et al., 2025).

From this perspective, it is more appropriate to argue that some adolescents—particularly those with higher gambling involvement—may rely on coping responses that reduce immediate emotional distress without directly addressing the stressor itself. Over time, this pattern may limit opportunities for learning from adverse experiences, correcting maladaptive beliefs, and developing more effective self-regulation skills. This interpretation is also consistent with recent prevention research. A contemporary systematic review of youth gambling prevention programs concluded that such interventions can improve coping, awareness, self-monitoring, problem-solving, and decision-making, and that many programs report short-term reductions in gambling frequency and severity. Accordingly, coping strategies should be regarded not only as correlates of gambling severity, but as modifiable targets for prevention and early intervention (Monreal-Bartolomé et al., 2023).

Furthermore, omitted variables would likely not operate in isolation, but rather by modulating the coping strategies already observed. Specifically, substance use may function as an immediate form of escape or self-regulation, reinforcing profiles characterized by emotional avoidance, passive waiting, and difficulty expressing distress, while weakening more protective strategies such as seeking social support or autonomy; this is consistent with the evidence linking avoidant coping to both problematic alcohol use and greater severity of addictive behaviors (Hahn et al., 2026). Similarly, family dynamics marked by conflict, low support, or poor communication may intensify this avoidant pattern, whereas a more supportive family environment could promote social support seeking, problem solving, and emotion regulation. In fact, prevention research suggests that interventions aimed at improving family dialog and coping skills are associated with better outcomes in adolescent and young adult problem gambling (Smith et al., 2025).

### Limitations and Further Lines of Research

The study’s findings are instrumental in developing targeted prevention programs within schools to mitigate the risk of GDs among adolescents. By equipping young people with healthy coping strategies, educators and mental health professionals can effectively address and reduce gambling-related issues. The research also highlights the importance of enhancing the skills of school staff in early identification of GD behaviors and promoting positive coping mechanisms. However, the study has limitations as it primarily focused on executive functions and coping strategies while overlooking other critical variables such as substance use, mental health issues, and family dynamics that contribute to gambling problems. Although a study based on an artificial neural network (ANN) provides predictive value in contexts where relationships between variables are not necessarily linear, its interpretation depends largely on the training data. To establish a more comprehensive artificial neural network for predicting and addressing these issues, future research must include a wider array of factors. Such an approach would facilitate better prevention strategies and limit adolescents’ engagement in gambling through new platforms and micropayments. While snowball sampling is useful for obtaining sensitive information when samples are difficult to reach and data collection costs need to be reduced, it may introduce bias and limit the representativeness of the sample. Therefore, it would be interesting to consider other sampling methodologies for future research. In addition, the extreme class imbalance (~0.1% prevalence) may also bias the model’s performance and limit its practical applicability. To sum up, given the severe class imbalance, the accuracy may overestimate model performance, and additional metrics such as sensitivity and F1-score should be considered.

## 5. Conclusions

In summary, the findings suggest that certain coping strategies are associated with gambling behaviors. While the neural network showed moderate overall performance, its limited ability to detect high-risk cases and the imbalance in the dataset highlight the need for further research with more representative samples and robust validation procedures. Future research should employ qualitative methods, such as in-depth interviews, to create supportive environments for women, thereby facilitating a more accurate assessment of their gambling behaviors. Additionally, a comprehensive analysis of the types of gambling games preferred by different genders will provide valuable insights into the social constructs surrounding gambling. It is essential to acknowledge the potential stigma that may deter women from expressing gambling-related issues, which could skew the understanding of addiction prevalence across genders. This multifaceted approach will contribute to a deeper understanding of gambling behavior.

## Figures and Tables

**Table 1:** G-SAS results (%).

Risk Level	Score	Percentage
No problem	Non-players	83.99
Low risk	0	6.8
	1	3.3
	2	3.0
Moderate Risk	3	2.3
	4	0.2
	5	0.2
	6	0.1
	7	0
Problem Gambling	8−10	0.1

**Table 2:** Correlations between G-SAS criteria and coping strategies (EEC-M).

		Sig.		95% Confidence Intervals
		(Bilateral)		(Bilateral)
				Lower Upper
Troubleshooting	0.030	0.721	−1.31	3.72
Search for social support	−0.124	0.135	−5.521	−0.042
Waiting	−0.303 **	0.00	1.021	6.360
Religion	0.284 **	0.00	−1.173	3.354
Emotional avoidance	0.309 **	0.00	0.599	6.453
Search for professional support	0.109	0.189	−4.163	0.657
Aggressive reaction	0.318 **	0.00	−1.501	1.880
Cognitive avoidance	0.115	0.164	−0.402	2.825
Positive reassessment	0.078	0.342	0.254	3.751
Expression of coping difficulty	0.267 **	0.01	−0.039	2.340
Denial	0.258 **	0.02	−0.156	1.211

The results with ** are statistically significant.

**Table 3:** Artificial neural network classification.

			Forecast	
Sample	Observed	0	1	Percent Correct
Training	0	99	5	95.2%
	1	17	3	15.0%
	Percentage Global	93.5%	6.5%	82.3%
Testing	0	51	1	98.1%
	1	8	1	11.1%
	Percentage Global	96.7%	3.3%	85.2%
Reserved	0	12	0	100.0%
	1	1	0	0.0%
	Percentage Global	100.0%	0.0%	92.3%

**Table 4:** Analysis of the importance of independent variables for the neural model.

	Importance	Normalized Importance
Sex	0.093	86.4%
Age	0.042	39.2%
Troubleshooting	0.040	37.1%
Search for social support	0.054	50.05%
Waiting	0.018	16.60%
Religion	0.013	12.4%
Emotional avoidance	0.009	8.1%
Search for professional support	0.061	57.0%
Aggressive reaction	0.024	22.1%
Cognitive avoidance	0.061	56.7%
Positive reassessment	0.080	73.90%
Expression of coping difficulty	0.020	18.80%
Denial	0.087	80.80%
Autonomy	0.108	100%

## Data Availability

The original contributions presented in this study are included in the article. Further inquiries can be directed to the corresponding author.
